# Supplementary material for: Nordic Seas polynyas and their role in preconditioning marine productivity during the Last Glacial Maximum
Source: Nat Commun. 2018 Sep 27;9:3959. doi: 10.1038/s41467-018-06252-8 (PMC6160467; doi:10.1038/s41467-018-06252-8)
Supplement: Supplementary file 1 — Supplementary Information [file 41467_2018_6252_MOESM1_ESM.pdf]

SUPPLEMENTARY INFORMATION

Nordic Seas polynyas and their role in preconditioning life during the Last Glacial Maximum

Knies et al.

Supplementary Table 1: Details of studied core locations displayed in Figure 1 and 7.

Station	Latitude N	Longitude E	Location	Water depth in meter	Reference
GS14-190-01PC	71.4755	16.1650	SW Barents Sea	949	this study
33-GC08	71.4753	16.1650	SW Barents Sea	947	this study
PS2138-1	81.5377	30.8761	N Barents Sea	995	Knies, et al. [1]
MSM05/5_712-2	78.9157	6.7672	W Svalbard	1389	Müller and Stein [2]
PS2446-4	82.3855	40.9013	N Barents Sea	2022	Knies, et al. [1]
PS2837-5	81.2442	2.3903	NW Svalbard	1042	Müller, et al. [3]

Supplementary Table 2: Sedimentological and geochemical raw data of GC14-190-01PC.

Age (cal. ka BP)	Depth original (cm)	Depth corrected (cm)	δ18O N. globoquadrina sin.	No. Planktic Forams/g >125µm)
15.25	25.00	5	4.66	
15.45	33.00	13	4.29	2152
15.69	41.00	21	4.14	2752
15.97	51.00	31	4.04	
16.23	61.00	41	3.66	12086
16.41	65.50	45.5	3.28	
16.64	71.00	51	3.08	
16.71	72.50	52.5	2.44	
16.80	74.50	54.5	3.44	
16.84	75.50	55.5	3.53	
17.03	79.50	59.5	2.39	
17.10	81.00	61	2.68	6946
17.16	82.50	62.5	3.45	
17.26	84.50	64.5	3.46	
17.30	85.50	65.5	3.47	
17.35	86.50	66.5	3.45	
17.56	91.00	71	4.43	
17.68	93.50	73.5	3.95	
17.72	94.50	74.5	3.71	
17.97	104.10	84.1	5.19	2336
18.19	114.10	94.1	5.17	
18.44	125.10	105.1	5.17	328
18.58	131.10	111.1	4.71	
18.86	143.10	123.1	5.07	1963
19.10	153.10	133.1	5.15	
19.28	163.10	143.1	5.22	1328
19.43	173.10	153.1	5.07	
19.55	182.10	162.1	5.13	3296
19.73	194.10	174.1	5.22	
19.88	204.00	184	5.25	2684
20.03	215.00	195	5.14	
20.22	225.00	205	5.12	5260
20.40	234.00	214	5.20	
20.61	245.00	225	5.22	2853
20.81	255.00	235	5.11	
21.06	268.00	248	5.17	11349
21.20	275.00	255	5.12	
21.39	285.00	265	5.13	4491
21.57	295.00	275	4.95	
21.74	305.00	285	4.99	3808
21.90	315.00	295	4.90	
22.09	325.00	305	4.86	3733
22.28	335.00	315	4.90	
22.48	345.00	325	4.94	6224
22.66	355.00	335	4.91	
22.82	365.00	345		0
22.98	375.00	355	4.82	
23.17	388.00	368	4.98	130
23.30	396.20	376.2	4.80	
23.46	407.20	387.2	4.85	13896
23.58	415.20	395.2	4.85	
23.71	423.20	403.2	4.78	7940
23.83	431.20	411.2	4.81	
23.96	440.20	420.2	4.85	25680

24.13	451.20	431.2	4.84	
24.27	461.20	441.2	5.02	5072
24.39	469.20	449.2	4.90	
24.54	478.20	458.2	4.80	13696
24.75	488.20	468.2	4.86	
24.91	496.00	476	4.78	2864
25.09	505.00	485	4.63	
25.29	515.00	495	4.37	17376
25.49	525.00	505	4.81	
25.64	533.00	513	4.78	9344
25.82	543.00	523	4.79	
26.01	553.00	533	4.74	3416
26.32	563.00	543	4.92	
26.56	570.00	550	4.78	1760
26.99	583.00	563	4.76	
27.34	593.60	573.6	4.73	7648
27.68	603.60	583.6	4.80	
27.94	611.60	591.6	4.63	16155
28.29	622.60	602.6	4.41	
28.60	631.60	611.6	4.57	5424
28.97	642.60	622.6	4.30	
29.31	652.60	632.6	3.98	1384
29.58	662.60	642.6	3.49	
29.85	672.60	652.6	3.82	32
30.12	682.60	662.6	4.98	
30.31	689.60	669.6	4.56	1508
30.63	701.40	681.4	4.63	

Age (cal. ka BP)	Depth original (cm)	Depth corrected (cm)	Grain size >250 (wt.%)	IP25 (µg/g sed.)
15.57	37	17	2.6	0.0000
15.91	49	29	1.2	0.0006
16.12	57	37	1.9	0.0005
16.35	64	44	3.8	0.0005
16.82	75	55	8.1	0.0000
17.14	82	62	0	0.0010
17.61	92	72	0.4	0.0006
17.81	97	77	4.6	0.0000
18.01	106	86.1	4.9	0.0017
18.23	116	96.1	1.8	0.0019
18.47	126	106.1	0	0.0018
18.74	138	118.1	4.6	0.0029
18.95	147	127.1	2.8	0.0025
19.18	157	137.1	2.6	0.0025
19.35	168	148.1	7.3	0.0023
19.50	178	158.1	10.6	0.0028
19.63	187	167.1	24.6	0.0035
19.78	197	177.1	1.6	0.0024
19.85	202	182	1.2	0.0025
19.99	212	192	0.01	0.0039
20.17	222	202	1.9	0.0022
20.36	232	212	0.7	0.0053
20.55	242	222	1.1	0.0045
20.75	252	232	1	0.0064
20.94	262	242	0	0.0048
21.14	272	252	0.1	0.0016
21.34	282	262	7.1	0.0037
21.45	288	268	0	0.0051
21.59	296	276	3.7	0.0032
21.75	306	286	9.6	0.0013
21.92	316	296	2.8	0.0027
22.11	326	306	6.5	0.0028
22.30	336	316	5.7	0.0033
22.50	346	326	7.8	0.0023
22.68	356	336	0.6	0.0025
22.84	366	346	1.6	0.0022
22.99	376	356	0	0.0015
23.14	386	366	0	0.0026
23.30	396	376.2	8.7	0.0040
23.52	411	391.2	4.9	0.0022
23.67	421	401.2	2.1	0.0074
23.83	431	411.2	4.9	0.0064
23.97	441	421.2	15.1	0.0053
24.13	451	431.2	6.8	0.0031
24.27	461	441.2	8.7	0.0030
24.39	469	449.2	8.2	0.0032
24.56	479	459.2	5.4	0.0026
24.77	489	469.2	5.7	0.0042
24.89	495	475	3.4	0.0047
25.09	505	485	6.7	0.0012
25.29	515	495	2.1	0.0000
25.49	525	505	0.6	0.0032
25.64	533	513	17.8	0.0019
25.82	543	523	0.9	0.0018
26.01	553	533	2.2	0.0000
26.32	563	543	0.1	0.0027

26.59	571	551	2.4	0.0020
27.06	585	565	0.01	0.0024
27.41	596	575.6	2.3	0.0007
27.74	606	585.6	2.2	0.0007
28.07	616	595.6	1.4	0.0026
28.39	626	605.6	0.5	0.0006
28.74	636	615.6	0.6	0.0031
29.07	646	625.6	3.3	0.0020
29.40	656	635.6	6.3	0.0011
29.66	666	645.6	2.7	0.0008
29.93	676	655.6	0.4	0.0000
30.20	686	665.6	0.4	0.0000
30.41	693	673.4	2.8	0.0016
30.68	703	683.4	1.9	0.0015
30.88	710	690.4	3.4	0.0019

Age (cal. ka BP)	Depth original (cm)	Depth corrected (cm)	Dinosterol (µg/g sed.)
15.61	38.50	18.5	0.0528
15.73	42.50	22.5	0.0785
15.88	47.80	27.8	0.0763
15.95	50.50	30.5	0.0516
16.30	62.80	42.8	0.0425
16.35	64.00	44	0.0376
16.41	65.50	45.5	0.0178
16.66	71.50	51.5	0.0117
16.82	75.00	55	0.0108
16.88	76.30	56.3	0.0031
17.03	79.50	59.5	0.0050
17.14	82.00	62	0.0084
17.21	83.50	63.5	0.0095
17.30	85.50	65.5	0.0163
17.58	91.50	71.5	0.0111
17.61	92.00	72	0.0050
17.73	94.80	74.8	0.0045
17.81	97.00	77	0.0039
17.99	104.80	84.8	0.0082
18.20	114.50	94.5	0.0321
18.23	116.10	96.1	0.0461
18.29	118.50	98.5	0.0308
18.36	121.50	101.5	0.0310
18.43	124.50	104.5	0.0309
18.47	126.10	106.1	0.0372
18.50	127.50	107.5	0.0234
18.57	130.50	110.5	0.0242
18.64	133.80	113.8	0.0295
18.74	138.10	118.1	0.0272
18.85	142.50	122.5	0.0359
18.95	147.10	127.1	0.0581
19.04	150.50	130.5	0.0538
19.15	155.50	135.5	0.0544
19.22	159.30	139.3	0.0657
19.35	168.10	148.1	0.0490
19.42	172.50	152.5	0.0667
19.49	177.50	157.5	0.0927
19.53	180.50	160.5	0.0667
19.65	188.50	168.5	0.0607
19.77	196.50	176.5	0.0700
19.81	199.50	179.5	0.0684
19.99	212.00	192	0.0647
20.17	222.00	202	0.0530
20.36	232.00	212	0.0456
20.55	242.00	222	0.0688
20.75	252.00	232	0.0758
20.94	262.00	242	0.0722
21.14	272.00	252	0.0566
21.34	282.00	262	0.0417
21.45	288.00	268	0.0385
21.55	293.50	273.5	0.0441
21.59	296.00	276	0.0518
21.61	297.50	277.5	0.0456
21.69	302.50	282.5	0.0603
21.77	307.50	287.5	0.0518
21.90	314.50	294.5	0.0717

21.97	318.50	298.5	0.0663
22.04	322.50	302.5	0.0464
22.16	328.50	308.5	0.0517
22.24	332.50	312.5	0.0675
22.30	336.00	316	0.0560
22.43	342.50	322.5	0.0532
22.52	347.50	327.5	0.0436
22.62	352.50	332.5	0.0481
22.72	358.50	338.5	0.0418
22.84	366.00	346	0.0272
22.91	370.50	350.5	0.0509
22.97	374.50	354.5	0.0369
23.06	380.50	360.5	0.0333
23.11	383.50	363.5	0.0453
23.17	387.50	367.5	0.0347
23.30	396.20	376.2	0.0806
23.52	411.20	391.2	0.0680
23.67	421.20	401.2	0.0731
23.83	431.20	411.2	0.0686
23.97	441.20	421.2	0.0577
24.13	451.20	431.2	0.0502
24.27	461.20	441.2	0.0747
24.39	469.20	449.2	0.0446
24.56	479.20	459.2	0.0892
24.77	489.20	469.2	0.0655
24.89	495.00	475	0.1050
25.09	505.00	485	0.0654
25.64	533.00	513	0.0144
25.82	543.00	523	0.0074
26.32	563.00	543	0.0570
26.59	571.00	551	0.0581
27.06	585.00	565	0.0707
27.41	595.60	575.6	0.0441
27.74	605.60	585.6	0.0475
28.07	615.60	595.6	0.0606
28.74	635.60	615.6	0.0628
29.07	645.60	625.6	0.0549
29.40	655.60	635.6	0.0193
29.66	665.60	645.6	0.0230
29.93	675.60	655.6	0.0237
30.20	685.60	665.6	0.0324
30.41	693.40	673.4	0.0258

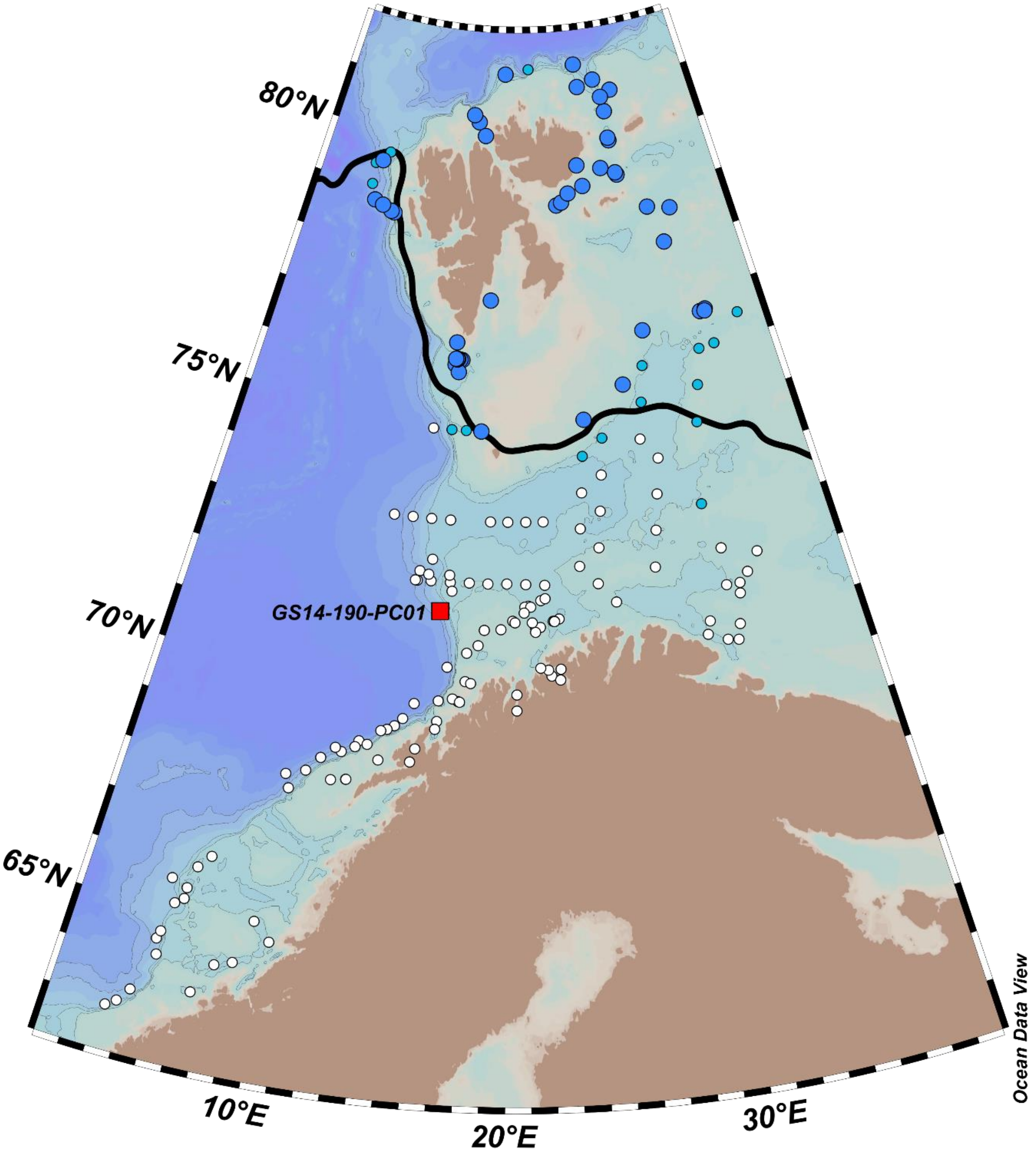

Supplementary Figure 1: Map showing the absence and presence of IP_{25} in surface samples (0-1 cm) in the Norwegian and Barents Sea[4] as well as the difference to the mean concentration in all IP_{25} containing sediments (large blue circles are above the mean IP_{25} value; small blue circles are below the mean IP_{25} value). The location of piston core GS14-190-01PC is marked. Lateral transport by bottom currents along-slope or down-slope do not influence the surface pattern of IP_{25} concentration. Its concentration is primarily controlled by the presence or absence of sea ice and vertical transport of particulate organic material through the water column.

Supplementary References

1 Knies, J., Kleiber, H. P., Matthiessen, J., Muller, C. & Nowaczyk, N. Marine ice-rafted debris records constrain maximum extent of Saalian and Weichselian ice-sheets along the northern Eurasian margin. *Global and Planetary Change* **31**, 45-64, doi:10.1016/s0921-8181(01)00112-6 (2001).

2 Müller, J. & Stein, R. High-resolution record of late glacial and deglacial sea ice changes in Fram Strait corroborates ice-ocean interactions during abrupt climate shifts. *Earth and Planetary Science Letters* **403**, 446-455, doi:10.1016/j.epsl.2014.07.016 (2014).

3 Müller, J., Masse, G., Stein, R. & Belt, S. T. Variability of sea-ice conditions in the Fram Strait over the past 30,000 years. *Nature Geoscience* **2**, 772-776, doi:10.1038/ngeo665 (2009).

4 Köseoğlu, D. *et al.* Complementary biomarker-based methods for characterising Arctic sea ice conditions: A case study comparison between multivariate analysis and the PIP25 index. *Geochimica et Cosmochimica Acta* **222**, 406-420 (2018).
